# Magnesium Is a Key Player in Neuronal Maturation and Neuropathology

**DOI:** 10.3390/ijms20143439

**Published:** 2019-07-12

**Authors:** Ryu Yamanaka, Yutaka Shindo, Kotaro Oka

**Affiliations:** 1 Center for Biosciences and Informatics, School of Fundamental Science and Technology Graduate School of Science and Technology, Keio University, Yokohama, Kanagawa 223-8522, Japan; 2 Faculty of Pharmaceutical Sciences, Sanyo-Onoda City University, Sanyo-Onoda, Yamaguchi 756-0884, Japan; 3 Graduate Institute of Medicine, College of Medicine, Kaohsiung Medical University, Kaohsiung City 80708, Taiwan; 4 Waseda Research Institute for Science and Engineering, Waseda University, 2-2 Wakamatsucho, Shinjuku, Tokyo 162-8480, Japan

**Keywords:** magnesium, neuron, differentiation, neural network maturation, synaptogenesis, intracellular signal, neurodegenerative disease

## Abstract

Magnesium (Mg) is the second most abundant cation in mammalian cells, and it is essential for numerous cellular processes including enzymatic reactions, ion channel functions, metabolic cycles, cellular signaling, and DNA/RNA stabilities. Because of the versatile and universal nature of Mg^2+^, the homeostasis of intracellular Mg^2+^ is physiologically linked to growth, proliferation, differentiation, energy metabolism, and death of cells. On the cellular and tissue levels, maintaining Mg^2+^ within optimal levels according to the biological context, such as cell types, developmental stages, extracellular environments, and pathophysiological conditions, is crucial for development, normal functions, and diseases. Hence, Mg^2+^ is pathologically involved in cancers, diabetes, and neurodegenerative diseases, such as Parkinson’s disease, Alzheimer’s disease, and demyelination. In the research field regarding the roles and mechanisms of Mg^2+^ regulation, numerous controversies caused by its versatility and complexity still exist. As Mg^2+^, at least, plays critical roles in neuronal development, healthy normal functions, and diseases, appropriate Mg^2+^ supplementation exhibits neurotrophic effects in a majority of cases. Hence, the control of Mg^2+^ homeostasis can be a candidate for therapeutic targets in neuronal diseases. In this review, recent results regarding the roles of intracellular Mg^2+^ and its regulatory system in determining the cell phenotype, fate, and diseases in the nervous system are summarized, and an overview of the comprehensive roles of Mg^2+^ is provided.

## 1. Introduction

Magnesium (Mg) is the second-most abundant cation following potassium in mammalian cells, and it is essential for numerous cellular processes, including enzymatic reactions, ion channel functions, metabolic cycles, and cellular signaling, as well as the stability of biomolecules, such as RNA, DNA, and proteins [1,2,3]. Mg plays a special role in biochemistry because of its smallest ionic radius, highest charge density, and largest hydrated radius, and it coordinates six oxygen atoms in its first coordination shell [3,4]. Mg^2+^ links together two phosphate groups in a macromolecule, which is responsible for the folding of biomolecules, such as enzymes and DNA/RNA. The inorganic chemistry of Mg plays a key role in the first chemical processes, which lead to the origin of life, i.e., ribozymes, and the early evolution of life [5,6,7]. Because of the essential roles of Mg^2+^, fundamental requirements of Mg^2+^ for biological processes seem to pose constraints on the evolution of cells and organisms. This fundamental nature of Mg^2+^ in life leads to the versatility and universality of the roles of Mg^2+^ in living systems. The homeostasis of intracellular Mg^2+^ is physiologically linked to cell growth, differentiation, energy metabolism, and cell death via the control of enzymatic activities, channel openings, DNA/RNA stability, and cellular stress [1,2,3]. Hence, it is crucial to regulate the Mg^2+^ concentration ([Mg^2+^]) within optimal levels according to the cell type and environment not only for normal functions and development but also prevention of diseases. In fact, disorder of Mg^2+^ homeostasis is involved in cancer, diabetes, and neurodegenerative diseases, such as Parkinson’s disease (PD), Alzheimer’s disease (AD), and demyelination [1,2,3,8,9]. The significance of intracellular Mg^2+^ is universal and fundamental at the molecular level, but its multiple and complex functions lead to its cell-type-specific roles. Previous studies suggested that, especially in the nervous system, Mg^2+^ plays specific roles in development, brain functions, and diseases [2,3,10]. Because of the contradictory observations, e.g., Mg^2+^ is trophic or toxic, an activator or an inhibitor, increased or decreased in the pathology of several diseases, the roles of intracellular Mg^2+^ and its regulatory system are controversial. In this review, the aim is to summarize the findings regarding the roles of intracellular Mg^2+^ and its regulatory system for determining cell phenotypes and fates in the nervous system and to provide an overview of the comprehensive roles of Mg^2+^ in neuro(patho)physiology.

## 2. Magnesium Homeostasis in the Brain

### 2.1. Magnesium Homeostasis in the Nervous System

**Figure 1:**
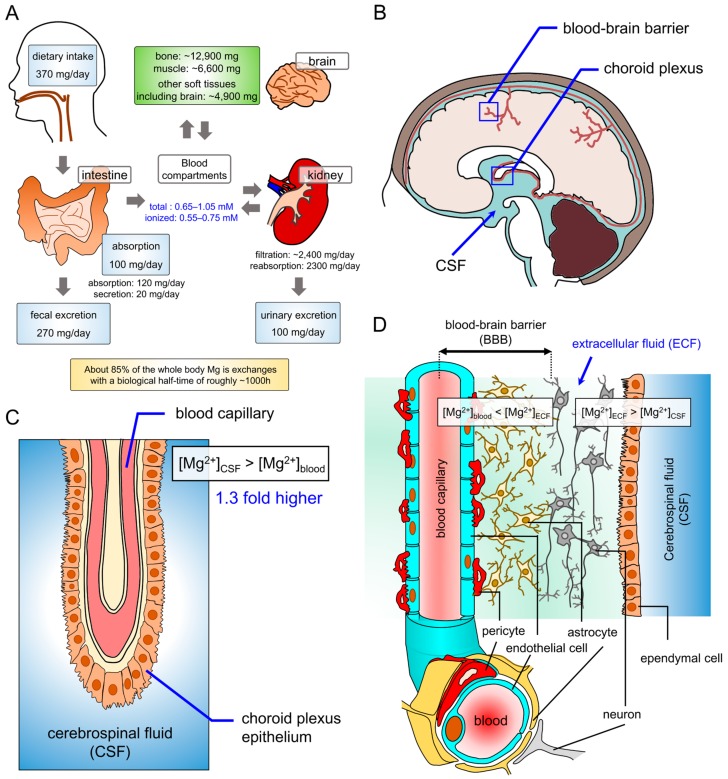
Magnesium homeostasis in whole body and brain. (**A**) Magnesium metabolism of the human body. (**B**) Choroid plexus and blood–brain barrier in the human brain. (**C**) The enlarged image of the boxed region in panel **B**, i.e., choroid plexus. The enlarged image of the boxed region in the panel **B**, i.e., blood–brain barrier (BBB). The gradient of [Mg^2+^] between blood and cerebrospinal fluid (CSF). (**D**) The structure of BBB at cellular levels and the comparison of [Mg^2+^] between blood, extracellular fluid (ECF) and CSF.

Mg^2+^ from daily intake is absorbed in the intestine, and it is reabsorbed by the proximal tubule (10–20%) and the thick ascending limb of Henle’s loop (50–70%) [3]. In the entire body, the majority of Mg^2+^ is accumulated in the bone, muscle, and soft tissues (Figure 1). The serum [Mg^2+^] ranges between 0.5 and 1.05 mM [11,12], the values of which reflect only 1% of the total content of Mg^2+^ in the body of a healthy person [3]. Even under severely Mg^2+^-depleted conditions, up to 80% of dietary Mg^2+^ can be absorbed [13], and most of Mg^2+^ in whole body exchanges at a very slow rate with biological half-time of 1000 hours [14]. Even in such conditions, the [Mg^2+^] in the serum is maintained within the normal range [3]. Extracellular fluid (ECF) in the central nervous system (CNS) is separated from the blood circulation by the blood–brain barrier (BBB). The BBB comprises endothelial cells of brain capillaries and allows passage of nutrients and electrolytes for the maintenance of ECF homeostasis. Because neuronal and glial cells are closely located with a distance of 20 to 50 nm and the volume of extracellular space is quite small in brain unlike the other organs [15,16,17], concentrations of the ECF components is greatly fluctuating. Thus, the BBB actively transport several molecules for the ECF homeostasis [17]. The [Mg^2+^] in ECF is maintained within a greater level compared with that of plasma or cerebrospinal fluid (CSF) [18]. The gap provides evidences for the active transport of Mg^2+^ in BBB. The in vitro BBB model of human brain endothelial cells, several functionally active Mg^2+^ transporters are expressed, such as transient receptor potential melastatin 7 (TRPM7) and MagT1 [19]. However, little has been revealed about the mechanism of Mg^2+^ transport in BBB. As most of the researches about Mg^2+^ absorption and excretion have focused on the small intestine and kidney [3,17], further investigation is required on how similar and different such organs are to CNS. In addition, the gap-junction-mediated cytosolic [Mg^2+^] ([Mg^2+^]_cyto_) regulates the circadian rhythm of BBB permeability in Drosophila, indicating that the intracellular [Mg^2+^] of BBB affects the neuronal environment in the brain [20]. The cerebrospinal fluid (CSF) fills and surrounds the brain and the spinal cord and exists at about 100 to 150 mL in the normal adult human body [21]. CSF functions as a mechanical barrier, and it is produced by the dialysis of blood and active transport of molecules, such as nutrients, hormones, metal ions and metabolites across the ependymal cells in the choroid plexus at a rate of 0.2 to 0.7 mL per minute [22]. The [Mg^2+^] of CSF is greater than that of blood [23,24], indicating that Mg^2+^ is actively transported from the blood into CSF [17]. The [Mg^2+^] of ventricular CSF is higher and more sensitive to changes in [Mg^2+^] of plasma than that of lumber CSF in cow [25]. The alteration of [Mg^2+^] of CSF correlates with the extracellular [Mg^2+^] around neurons, which affects neural activities. Thus, the [Mg^2+^] of CSF is closely related to various brain functions [26,27]. In particular, the [Mg^2+^] of CSF and cognitive functions have been reported to exhibit a positive correlation [28,29,30,31,32]. In addition, the intracellular [Mg^2+^] of erythrocytes significantly correlates with the [Mg^2+^] of CSF in the hippocampus, and further with the hippocampal synapse density and recognition and memory performance [33,34], suggesting that [Mg^2+^]_cyto_ of erythrocytes is a good index of recognition and memory. These facts revealed that Mg^2+^ homeostasis in the human body is a key factor in brain functions, especially synaptic connectivity.

### 2.2. Mg^2+^ Transport in Neurons

Mg^2+^ is the most abundant divalent cation in cells, and the total [Mg^2+^] ranges between 17 and 20 mM in mammalian cells [1,2]. The gaps between the [Mg^2+^]_cyto_ and extracellular free Mg^2+^ concentration ([Mg^2+^]_ex_) are maintained within less than twofold (10,000- and 20,000-fold for Ca^2+^ and Zn^2+^, respectively) [1,35,36,37]. As the resting membrane potential of neurons is about −70 mV, if [Mg^2+^]_cyto_ is at the electrochemical equilibrium, then its resting concentration should be 50 mM [37]. Yet, even under the Mg^2+^-mobilized condition, only a slight change in [Mg^2+^]_cyto_ is observed, within about twofold [38,39,40]. Against the electrochemical gradient, cells exhibit several mechanisms to physiologically maintain intracellular [Mg^2+^] within a narrow range under resting or stimulated conditions [1,3,37]. Intracellular Mg^2+^ is regulated through a balance of influx, efflux, and the amount of stored intracellular Mg^2+^ [1,3,37], and it is fully exchanged with plasma Mg^2+^ within 3 to 4 hours [12]. The energy required for the transport of Mg^2+^ is several times greater than that required for the transport of other cations [41] because Mg^2+^ binds water molecules more tightly than other cations due to its highest charge density. As Mg^2+^ exhibits the largest hydrated radius (0.428 nm) and the smallest ionic radius (0.072 nm), the volume change between hydrated and ionic Mg^2+^ is almost 400-fold (Na^+^ and Ca^2+^: ∼25-fold or K^+^: 4-fold) [42,43]. Thus, any protein transporting Mg^2+^ must be capable of initially interacting with the large cation [42]. Furthermore, assuming that Mg^2+^ is transported in its ionic form similar to other cations, dehydrated Mg^2+^ passes through quite a small pore. Together, Mg^2+^ transporting molecules must contain both physically large initial binding sites and small pores. Because of these unique characteristics of Mg^2+^, the structure and mechanism of Mg^2+^ transporters have not been completely revealed. Although several Mg^2+^-transporting proteins are identified in mammalian cells [1,3,37,44,45], the association of these Mg^2+^-transporting systems with the neuro(patho)physiology has not been investigated much.

### 2.3. Mg^2+^ Distribution of Cells

**Figure 2:**
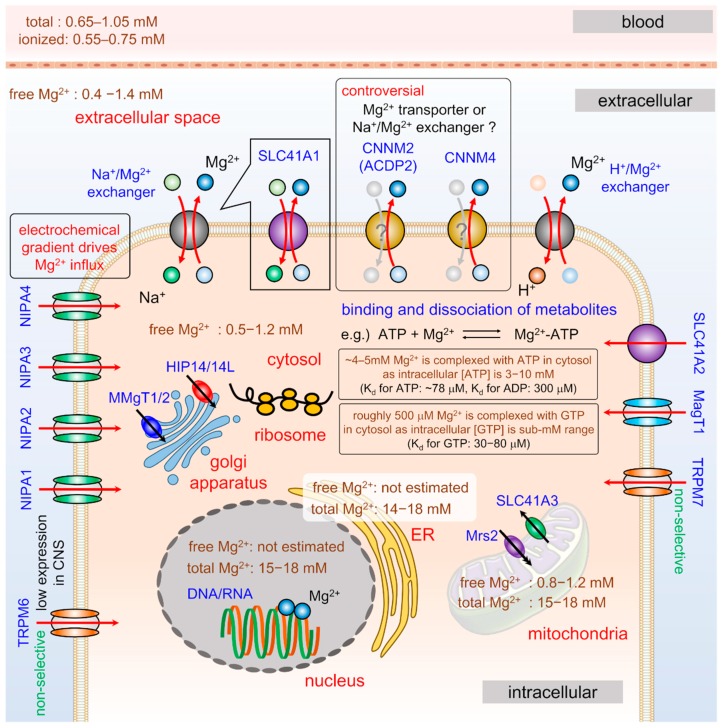
Intracellular Mg^2+^ distribution and machinery for Mg^2+^ regulation: The intracellular Mg^2+^ content is regulated by the balance of influx, efflux, and the intracellularly stored amount. Major storages for intracellular Mg^2+^ are the nuclei, mitochondria, ERs, and the ribosome. Cytosolic Mg^2+^ is bound to phosphometabolites, such as ATP. NIPA1, NIPA2, NIPA3, NIPA4, MagT1, TRPM6, TRPM7 and SLC41A2 contributes the uptake of extracellular Mg^2+^. Na^+^/Mg^2+^ and H^+^/Mg^2+^ exchangers contribute the Mg^2+^ efflux. Some studies support that SLC41A1, CNNM2 and CNNM4 functions as Mg^2+^ exchangers. MMgT1/2 and HIP14/14L is localized at golgi apparatus, and Mrs2 and SLC41A3 is localized at mitochondria. Abbreviations: ER—endoplasmic reticulum; ATP—adenosine 5’-triphosphate; TRPM6—transient receptor potential melastatin 6; TRPM7—transient receptor potential melastatin 7; CNNM2 or ACDP2—cyclin M2; CNNM4—cyclin M4.

Nuclei, mitochondria, and endoplasmic or sarcoplasmic reticulum (ER/SR) compartmentalize intracellular Mg^2+^ with total concentrations ranging between 15 and 18 mM [1,37]. In the lumen of these organelles, only a small fraction of Mg^2+^ is free, and almost all of the Mg^2+^ is complexed with negatively charged biomolecules in each compartment because of its positive charge [2,3,37] (Figure 2).

#### 2.3.1. Cytosol

In the cytosol, Mg^2+^ is complexed to a broad spectrum of biomolecules, such as phosphonucleotides, phosphometabolites, and Mg^2+^-binding proteins [1,37]. In particular, adenosine 5′-triphosphate (ATP) is the major intracellular pool for Mg^2+^ because of its abundance (on the order of millimolar concentrations) and high binding affinity (K_d_ of ~78 µM) [1,37,46]. The Mg^2+^ buffering contributes to the maintenance of free [Mg^2+^]_cyto_ within a narrow range. In such intracellular environments, some biological stimuli induce slight but significant changes in [Mg^2+^]_cyto_ [38,47,48,49]. Even when the changes in intracellular [Mg^2+^] is apparently slight, the contents and distributions of Mg-complexed biomolecules may change dramatically under the conditions in which Mg^2+^-buffering molecules are abundant. Thus, a slight fluctuation of intracellular [Mg^2+^] can impact on cellular processes more than expected.

#### 2.3.2. Nuclei

Nuclear [Mg^2+^] ([Mg^2+^]_nuc_) considerably varies depending on the physiological conditions [50]. In nuclei, chromatin, nucleic acids, and free nucleotides require counterions for the neutralization of their negative charges [1,2,37]. K^+^ and Mg^2+^ are strong candidate cations for the neutralization of charges because of the low intracellular concentrations of Na^+^ and Ca^2+^. Furthermore, Mg^2+^ wins in the competition of K^+^ for binding to the negatively charged molecules, such as DNA in the nucleus, because it has more positive charges and a higher hydration energy [51]. For stabilizing the condensed state of DNA, a high concentration of counterions is required (1 to 2 M for distances between the DNA helix axes of 2 to 4 nm) [52]. Thus, in the nucleus, Mg^2+^ is localized at a spatially heterogeneous distribution. Recently, the genetically encoded fluorescent-protein-based Mg^2+^ sensor, which is named as Magnesium Ratiometric Indicator for Optical Imaging (MARIO), revealed that, after the breakdown of a nuclear envelope, [Mg^2+^]_nuc_ increases and peaks during the metaphase, and it gradually decreases during cytokinesis. The condensation of chromosomes by Mg^2+^ is required for cell mitosis [40,53]. In yeast, the deficit of Mg^2+^ intake interferes with the interphase microtubule organization and mitotic spindle formation [54]. In addition, some tumor cells contain higher levels of Mg^2+^ in the nucleus compared with normal cells [50,55,56]. As Mg^2+^ is known to affect cell proliferation over several decades [57,58], nuclear Mg^2+^ apparently plays an important role in cell division. In addition, Mg^2+^ is involved in genome regulation [59]. As Mg^2+^ affects the solubility of chromatin, and the chromatin surrounding the DNA affects gene expression, the Mg^2+^-fluctuation-dependent change in chromatin folding presumably affects genome regulation [60].

#### 2.3.3. Mitochondria

Mitochondria constitute a major cellular Mg^2+^ pool and contribute to the homeostatic regulation of intracellular Mg^2+^ [48,61,62,63]. Several physiological and pathological stimuli activate the release of mitochondrial Mg^2+^ into the cytosol, inducing a rapid, large decrease in the [Mg^2+^] in the mitochondrial matrix ([Mg^2+^]_mito_). Thus, mitochondria are a key player in the regulation of intracellular Mg^2+^ homeostasis [38,47,48,49,62]. As the [Mg^2+^]_mito_ is typically 0.8 to 1.2 mM [1,37,64,65], mitochondrial Mg^2+^ predominantly combines with adenine phosphonucleotides and Mg^2+^-binding proteins. In mammalian cells, the mitochondrial Mg^2+^ influx channel mitochondrial RNA splicing 2 (Mrs2) [48,66] and mitochondrial Mg^2+^ exporter SLC41A3 [67] are identified. Although the disruption of the mitochondrial membrane potential (ΔΨ_m_) triggers the release of Mg^2+^ from the mitochondria into the cytoplasm [47,49,61], the molecular mechanism for the release of mitochondrial Mg^2+^ has not been revealed.

Mitochondrial Mg^2+^ affects several mitochondrial functions: (1) mitochondrial energy metabolism, (2) apoptotic process, (3) mitochondrial Ca^2+^ homeostasis, and (4) mitochondrial DNA functions.

##### Mitochondrial Energy Metabolism

Mg^2+^ is required for a wide range of biochemical processes in mitochondrial energy metabolism. Mitochondrial Mg^2+^ homeostasis demonstrates the potential to regulate the rate of energy production according to energy demand. Activities of 2-oxoglutarate dehydrogenase (OGDH), which is the rate-limiting enzyme of the tricarboxylic acid (TCA) cycle, and several other enzymes are stimulated by Mg^2+^ [63,68]. In addition, Mg^2+^ contributes to the transport of ATP from the mitochondria to the cytoplasm, which is mediated by an ATP-Mg/P_i_ carrier [69]. The accumulation of ATP in mitochondria inhibits several enzymatic processes in the TCA cycle [70] and activities of the electron transport chain [71] in a negative feedback manner. The decrease of [Mg^2+^]_mito_ by the *Mrs2* knockdown affects the metabolome, especially reactions involved in the TCA cycle [48].

##### Apoptotic Process

Changes in [Mg^2+^]_cyto_ are observed in several apoptotic cells [49,62,72]. For instance, anoxia induces the increase of [Mg^2+^]_cyto_ via TRPM7 channels in hippocampal neurons [73]. Apoptotic stimuli triggers the release of Mg^2+^ from mitochondria in various cells, including neurons [47,49,61,62,74]. The upregulation *Mrs2* levels suppress apoptosis in gastric cancer cells [75]. The downregulation of the *Mrs2* level causes decreased ΔΨ_m_ and abnormal mitochondrial morphology [48]. Taken together, the accumulated mitochondrial Mg^2+^ through the Mrs2 channel exhibits protective effects against cellular stress. The activation of the mitochondrial ATP-sensitive potassium channel (mitoK_ATP_), which contributes to the protective effects of an ischemic precondition [76,77], triggers the release of mitochondrial Mg^2+^ [47]. *N*-methyl-4-phenylpyridinium iodide (MPP^+^) is an active metabolite of PD inducer 1-methyl-4-phenyl-1,2,3,6-tetrahydropyridine (MPTP) [78]. In MPP^+^-induced mitochondrial stress in the PD model, the cytosolic Mg^2+^ level after mobilization from the mitochondria and extracellular medium is correlated with the cell viability [39]. These studies revealed that stored and released Mg^2+^ in mitochondria attenuates the neurodegeneration. This is explained by the fact that Mg^2+^ inhibits the opening of the permeability transition pore (PTP), leading to the release of cytochrome c and consequently apoptosis [79,80]. On the other hand, the elevated [Mg^2+^]_mito_ [81] and decreased [Mg^2+^]_cyto_ [82] are observed in some models of the induction of apoptosis. Mg^2+^ stimulates the release of cytochrome c independently of the PTP [83,84]. These opposite observations in cellular apoptotic processes suggest the missing mechanism, and further work is required to associate the apoptosis process to the Mg^2+^ homeostasis.

##### Mitochondrial Ca^2+^ Homeostasis

Mitochondrial Ca^2+^ uptake plays various roles in vital signaling processes, such as bioenergetics, cell death, and sequestration of cytosolic Ca^2+^ transients [85]. Extramitochondrial Mg^2+^ suppresses the mitochondrial uptake of Ca^2+^ via the inhibition of the Ca^2+^ uniporter [85,86,87,88]. Thus, the intracellular homeostasis of Mg^2+^ affects Ca^2+^-signal-mediated mitochondrial functions, such as bioenergetics and apoptosis.

##### Mitochondrial DNA Functions

Mammalian mitochondria contain their own DNA (mtDNA), which encodes the essential components of the oxidative phosphorylation (OXPHOS) and RNA elements. The Mg^2+^ requirements for the DNA/RNA structure and protein synthesis lead to the possibility that mitochondrial Mg^2+^ affects mitochondrial DNA (mtDNA) functions, as well as processes of protein synthesis in mitochondria (described in detail in Chapter 5.3).

#### 2.3.4. Endo(sarco)plasmic Reticulum

ER/SR accumulates Mg^2+^ (its total [Mg^2+^] is estimated to be between 14 and 18 mM), and Mg^2+^ binds to ribonuclear proteins and phospholipids [37]. The concentration of Ca^2+^ ([Ca^2+^]) is near 100 nM in the cytosol, while [Ca^2+^] in the ER ranges between 100 and 800 µM [89]. As high levels of Ca^2+^ in the ER/SR interfere with accurate measurements using fluorescent probes with a low selectivity for Mg^2+^, [Mg^2+^] in ER/SR has not been reliably determined. ER plays central roles in Ca^2+^ signaling in cells, including neurons [90,91]. Ca^2+^ is released from the intracellular Ca^2+^ storage in the ER/SR via inositol-1,4,5-trisphosphate receptors (IP3R) and ryanodine receptors (RyR). Mg^2+^ functions as an intracellular inhibitor of IP3R [92] and RyR [93,94,95]. As IP3R or RyR-mediated Ca^2+^ signaling plays several roles in neuronal processes including neuronal development and plasticity [90,91,96], the interaction of Mg^2+^ with Ca^2+^ signaling can significantly affect neuro(patho)physiology [95]. The decrease of [Mg^2+^]_cyto_ by caffeine as a RyR activator through an as yet unidentified mechanism in myocytes [97] suggested the possibility that SR/ER is involved in intracellular Mg^2+^ signals. The accurate determination of [Mg^2+^] in the ER/SR without Ca^2+^ interference has been eagerly awaited.

#### 2.3.5. Ribosome

The ribosome is a complex molecular machine that serves as the site of protein synthesis, i.e., translation. Ribosomes comprise ribosomal RNA (rRNA) molecules and ribosomal proteins. A single 70S ribosome of *Escherichia coli* comprises more than 170 Mg^2+^ atoms [98], meaning that an entire pool of 70,000 ribosomes chelates at least 12 mM Mg^2+^ in a single cell [99,100]. Since Mg^2+^ plays crucial roles in the structural stability and/or catalytic activity of the ribosome, which cannot be replaced by the other cations [101,102,103,104], [Mg^2+^]_cyto_ is closely associated with its ribosome content [105,106]. Since the majority of the ribosomes are typically translating at the maximum capacity, the overall rate of protein synthesis is often determined by the rate of ribosome synthesis [100]. In brief, [Mg^2+^]_cyto_ is highly correlated with the rate of protein synthesis [107]. Furthermore, in ribosomes, Mg^2+^ deficits cause the loss of peptidyl transferase activity and subsequent ribosome disassembly [108] and lead to the reduction of translation [109]. Studies with prokaryotic cells reveal that low [Mg^2+^]_cyto_ promotes protein expressions for Mg^2+^ uptake and ATP reduction, consequently making Mg^2+^ available for translation to satisfy the cellular demands of Mg^2+^ [110,111,112,113]. The homeostatic systems of intracellular [Mg^2+^] are suggested to be evolutionarily conserved in eukaryotic cells [112]. In mammalian cells, the mechanistic target of rapamycin (mTOR) signaling regulates several growth-related processes, including ribosome biogenesis [114]. Intracellular Mg^2+^ physiologically enhances mTOR signaling activities [38,115]. Thus, Mg^2+^ apparently regulates protein synthesis through the effect on ribosomal functions via the mTOR pathway [115,116]. In neurons, ribosomes perform protein synthesis, and their functions are essential for dendritic growth and maintenance [117,118]. Therefore, deficits of protein synthesis in ribosome disturb neurodevelopment via the reduction of neuronal connectivity [117]. The deficits of protein synthesis related to various neurodegenerative diseases, including AD [119,120] and PD [121,122,123]. Hence, [Mg^2+^]_cyto_ affects the formation of a neural network via ribosome biogenesis.

## 3. Physiological Roles of Cellular Mg^2+^

**Figure 3:**
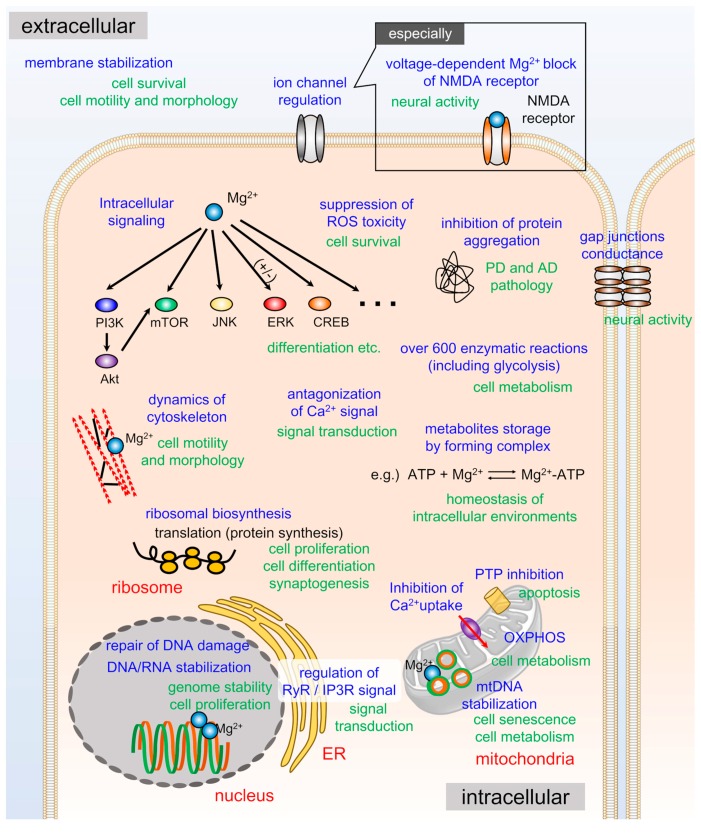
Overview of cell physiology of Mg^2+^. The intracellular Mg^2+^ plays versatile roles in neurons. Abbreviations: PI3K—phosphatidylinositol-3 kinase; mTOR—mechanistic target of rapamycin; JNK—c-Jun NH_2_-terminal kinase; ERK—extracellular signal-regulated kinase; CREB—cAMP response element binding; ROS—reactive oxygen species; NMDA—*N*-methyl-D-aspartate; AD—Alzheimer’s disease; PD—Parkinson’s disease; ATP—adenosine 5’-triphosphate; PTP—permeability transition pore; RyR—ryanodine receptor; IP3R—inositol-1,4,5-trisphosphate receptor; OXPHOS—oxidative phosphorylation; mtDNA—mitochondrial DNA.

In living cells, negative charges of biomolecules are in excess of the positive ones. Thus, inorganic metal ions, such as K^+^, Na^+^, Ca^2+^, and Mg^2+^, exceed the number of anions [52]. Although Ca^2+^ is a better competitor for binding to negative charges of biomolecules, the intracellular Ca^2+^ level is maintained at low levels under normal and resting conditions [35,36]. Hence, Mg^2+^ is the main candidate as a counterion for neutralizing negatively charged biomolecules, e.g., RNA/DNA, reactive oxygen species (ROS), and ATP, because of its abundance and multivalence [3,4]. The properties of Mg^2+^ for interacting with numerous biomolecules render versatile roles, e.g., a modulator of enzymatic activities, cell protection against cellular stress, channel regulation, and DNA/RNA stabilization (Figure 3). In addition, the interaction of Mg^2+^ with various biomolecules serves as an intracellular buffer and a storage system for maintaining the homeostasis of intracellular molecules. Hence, it is not surprising that dysregulation of Mg^2+^ homeostasis is tightly connected with several disease conditions, such as neurodegenerative disease, diabetes mellitus, and metabolic syndrome [2,3].

### 3.1. Biochemical Reactions in Cells

Mg^2+^ affects more than 600 enzymatic reactions, including energy metabolism, protein synthesis, and signal transduction [2,3,37,124]. In particular, the dependence of Mg^2+^ in cellular energy production has been documented over several decades. The requirement of Mg^2+^ in various enzymatic activities of glycolysis has been discussed in the textbook of biochemistry (pp. 228, [125]). All chemical reactions are governed by the laws of thermodynamics, that is, the Gibbs free-energy change (ΔG). The ΔG for the ATP hydrolysis varies from −28 to −34 kJ/mol, depending on the [Mg^2+^], because positively charged Mg^2+^ stabilizes ATP (Chapter 3 in [126], [127]). This fact implies that fluctuations of intracellular [Mg^2+^] affect all ATP-related biochemical reactions in cells; intracellular Mg^2+^ can function as a comprehensive regulator. Furthermore, intracellular Mg^2+^ competes not only with Ca^2+^ but also with protons or amines (–NH^2+^). Protons (H^+^) are typically present at concentrations of less than 10^−7^ M at pH 7 and bind to phosphate groups with a pKa of 6.5. Mg^2+^ is removed from ATP when the pH decreases to 6.0 [51], leading to significant effects on Mg^2+^-dependent reactions:

Mg · ATP + H^+^ ⇋ H · ATP + Mg^2+^

Each intracellular organelle has a characteristic concentration of protons ([H^+^]), indicating that Mg^2+^ impacts cellular biochemical reactions in an organelle-specific manner. Indeed, intracellular [Mg^2+^] rhythms dynamically tune cellular biochemistry in response to the metabolic demands throughout the daily cycle [128,129].

### 3.2. Intracellular Signaling

The free energies for the binding of ATP to protein kinase in the presence of Mg^2+^ are less than those in the absence of Mg^2+^ [130]. Mg^2+^ potentially enhances reactions of all protein kinases, that is, intracellular signal transduction. The function of intracellular Mg^2+^ as the second messenger has been controversial over decades [131,132]. In living cells, the electrochemical gradient of Mg^2+^ across the plasma membrane serves as a reservoir for signal generation. When Mg^2+^-permeable channels open in response to biological cues, Mg^2+^ influx should be initiated. In fact, although some biological stimuli induce intracellular Mg^2+^ mobilization [37,39,47,49,73], the regulatory mechanism of Mg^2+^ channels has not been revealed yet. The concentrations of Ca^2+^ and other cations fluctuate within several orders of magnitudes (from 10 nM to 100 µM in the case of Ca^2+^) in response to cellular events [35,36]. In contrast, intracellular [Mg^2+^] is maintained within the narrow and sub-millimolar ranges, which is considerably greater than that in the case of [Ca^2+^] [36]. Hence, Mg^2+^ is believed to function physiologically not as a biological switch but as a modulator. In 2011, the role of Mg^2+^ as the second messenger in immune cells was demonstrated by its three fundamental features as a second messenger: (1) Its levels increase rapidly in response to a biological stimulus. (2) It alters the rate of one or more cellular processes. (3) It exerts cell-type specific roles because it affects different complements of enzymes in a cell-type dependent manner [35,133]. This study revealed that the coupling of the Mg^2+^ influx with the activation of the cell-surface receptor is required for healthy functions of its downstream cellular responses. However, whether Mg^2+^ amplifies extracellular biological information has not been revealed. In developing neurons, gamma-aminobutyric acid (GABA)-induced Mg^2+^ mobilization enhances cAMP response element binding (CREB) and mTOR activities in a [Mg^2+^]_cyto_-dependent manner within the physiological dynamic range [38]. CREB signaling is essential in the several transcriptional events and the control of neuronal plasticity [134,135]. mTOR functions as an intracellular energy balance and metabolism regulator that controls protein synthesis, cell growth, and differentiation. Intracellular Mg^2+^ mobilization simultaneously activates CREB and mTOR signaling, and such signaling cooperatively enhances the maturation of neural networks. Notably, the [Mg^2+^]_cyto_ regulation of mTOR activities exhibits sigmoidal curves, indicating that Mg^2+^ functions as a cooperative signal amplifier [38,136]. In the case of the Ca^2+^ signal, the conformational changes of a Ca^2+^-binding protein, such as calmodulin, are evolutionarily conserved and play central roles in the switch-like regulation of biochemical reactions. Yet, with respect to Mg^2+^ signals, such a protein has not been identified. After all, the answer to the question of whether Mg^2+^ is a second messenger or not depends on the definition of the “second messenger.” At least, Mg^2+^ integrates and coordinates extracellular information and affects various cellular processes in probably all types of cells. [Mg^2+^]-dependent properties of such intracellular signaling are apparently more primitive and fundamental than are other intracellular signals considering the involvement of Mg^2+^ in the early emergence of life.

### 3.3. ROS Toxicity

Oxygen atoms function as electron acceptors in several metabolic processes. The majority of the oxygen consumed by biological systems is reduced to water, and it is converted to ROS. In this process, intermediate substances, such as hydroxy radicals (·OH) or superoxide anions (O^2−^), are toxic to the living body [137,138]. Free radical production increases in Mg^2+^-deficient animals [63,139,140]. Since O^2−^ may react with Mg^2+^ via an electron-transfer reaction, leading to the production of magnesium–oxygen species [4], Mg^2+^ suppresses the production of ROS in the various tissues, including the brain [39,140,141,142]. Hence, Mg^2+^ is considered to protect the living body from radicals because of its physicochemical properties.

### 3.4. Channel Regulation

Mg^2+^ regulates several ion channels [143]. Especially, in neurons, extracellular Mg^2+^ contributes to the activity control of one of the glutamate receptors, *N*-methyl-D-aspartate (NMDA) receptor, which plays crucial roles in neuronal functions [144,145]. In neurons at the resting membrane potential (−70 mV), Mg^2+^ blocks the NMDA receptor. When the membrane potential is increased to −30 mV via another glutamate receptor, AMPA receptor, activation and cation influx, Mg^2+^ block is relaxed, and the NMDA receptor is activated. With the decrease in extracellular [Mg^2+^], the membrane potential of neurons is weakly depolarized because of the relaxation of the Mg^2+^ block of the NMDA receptor, leading to hyperexcitability. As the NMDA receptor is involved in excitatory neurotransmission, neuroplasticity, and neuroexcitotoxicity, it plays an important role in developmental plasticity [146,147], learning and memory [32], and circadian clock rhythm [148].

### 3.5. DNA Protection and Genome Stability

Mg^2+^ is considerably required to maintain genomic stability. Mg^2+^ contributes to the maintenance of genome stability via two mechanisms: Its role as a cofactor in the DNA-repair-mechanism-related enzyme and as a competitive inhibitor of the DNA-damaging factor due to the binding of Mg^2+^ to DNA [59]. The positive charges of Mg^2+^ interact with the negative charges of the phosphate group of DNA, and it plays key roles in stabilizing the secondary and tertiary structures of DNA [149]. Mg(H_2_O)_6_^2+^, in which six water molecules are coordinated with Mg^2+^, forms a hydrogen bond with DNA. In the coordinated state, the denaturing agent cannot attack this site in DNA. Thus, intracellular Mg^2+^ protects the DNA from ROS [4]. In addition to stabilizing the DNA and chromatin structure, Mg^2+^ is an essential cofactor in almost all enzymatic processes involved in DNA [59]. The genetic information of DNA is replicated with high fidelity. Mg^2+^ plays a key role in DNA replication and repair [150]. DNA templates are copied by enzymatic processes involving DNA polymerases. In these processes, Mg^2+^ is required for replication with high fidelity [59,151]. DNA is continuously damaged by environmental and endogenous mutagens [152]. To maintain low mutation frequencies, cells have DNA repair systems, which require optimal [Mg^2+^] in multiple steps. Mg^2+^ contributes to the accurate transfer of genome information and resultant translation of functional protein.

## 4. Effects of Mg^2+^ on the Cellular Fate and Phenotype

### 4.1. Formation of Neural Networks and Synaptic Activities

Mg^2+^ is crucial for the growth and differentiation at the cellular and tissue levels [10]. In developing neurons, the neurotransmitter-induced increase in [Mg^2+^]_cyto_ mobilized from mitochondria stimulates mTOR activities in a [Mg^2+^]-dependent manner and facilitates the maturation of neural networks [38]. Typically, mTOR plays a central role in the regulation of the cellular metabolic state and protein synthesis in response to the high demand for growth and proliferation. In neurogenesis, the mTOR activation leads to dendritic arborization via the regulation of protein synthesis [153].

Another key player for Mg^2+^ mobilization in neuronal developments is the TRPM7 channel. The TRPM7 channel is composed of a non-selective divalent cation channel and protein kinase domain, which is called “chanzyme”. It is ubiquitously expressed and plays key roles in intracellular Mg^2+^ homeostasis [55,154,155,156,157,158,159], and the TRPM7 channel is regulated by intracellular signals and also its kinase domain [160,161,162]. Although TRPM7 is essential for early embryonic development [10,163,164,165], the contribution of Mg^2+^ influx through TRPM7 on the embryonic development remains controversial because TRPM7 acts as both channel and enzyme [10,164]. Several studies support the roles of kinase, which is responsible for the requirement of TRPM7 during embryogenesis [163,164,166]. The depletion of TRPM7 leads to disruption of the embryonic developments without affecting the uptake of Mg^2+^ in thymocytes [164]. These studies suggest a possibility that the contribution of TRPM7 in Mg^2+^ homeostasis is low in vivo or that disturbed Mg^2+^ homeostasis is compensated by other Mg^2+^ channels. Actually, cells possess several Mg^2+^ transporting systems [37,167], and Mg^2^^+^ homeostasis is robustly maintained through the compensation mechanism in vertebrates. In fact, the expressions of several Mg^2+^ transporters are simultaneously and dynamically changed in response to the cellular environments [168]. The properties of TRPM7 as the channel and kinase and compensatory maintenance of Mg^2+^ homeostasis causes the difficulties for revealing the roles of Mg^2+^ in animal developments. Recently, some groups have shown that inactivation of the TRPM7 channel in living mice results in impaired Mg^2+^ transport, supporting the notion that the TRPM7 channel indeed can function as important Mg^2+^ channel in vivo [165,169]. Moreover, careful analysis using TRPM7 mutants lacking kinase domain support that TRPM7-mediated Mg^2+^ influx is essential for embryonic developments in vertebrate [10,170].

TRPM7 is highly expressed in the tips of the growth cone [171]. The TRPM7-mediated Mg^2+^ influx in fibroblasts is required for lamellipodia formation, cell polarization, and directed cell migration [172]. In lymphocytes, the TRPM7-mediated Mg^2+^ influx is apparently associated with phosphoinositide 3-kinase (PI3K)/Akt/mTOR signaling [173,174]. Neurite outgrowth is dependent on the dynamic changes of the cytoskeleton within the growth cone, and it is energy-consuming and spatiotemporally controlled process. Thus, the entry of TRPM7-dependent Mg^2+^ presumably enhances the activation of mTOR to meet the energy demand for the neuronal network organization. In contrast, the TRPM7-mediated Ca^2+^ influx causes the generation of ROS and suppresses the polarization of hippocampal neurons. Physiological ROS production is required for the polymerization of the cytoskeleton [175]. ROS physiologically regulates cytoskeletal changes via the modification of cytoskeletal and cytoskeleton-regulated molecules for the appropriate formation of the neural network [176]. These facts suggest that the TRPM7 channel is crucial for the growth cone pathfinding via the prevention of the axonal overgrowth and connection with unwanted targets. In addition, TRPM7 responds to the membrane stretch and fluid shear force [177,178]. It seems the opposite roles of TRPM7-mediated Mg^2+^ and Ca^2+^ influxes cooperatively regulate the proper growth cone pathfinding in response to the extracellular mechanical stimuli.

In nervous systems, chemical synapse and electrical synapse functionally connect neuronal cells for the formation of neural networks. Electrical synapses are physically connected by channel proteins, forming gap junctions. [Mg^2+^]_cyto_ controls the strength of the electrical gap junctions [179,180]. Mathematical simulations revealed [Mg^2+^]_cyto_-dependent long-term plasticity through the regulation of electrical gap junctions [181]. Furthermore, chemical-synapse-mediated neural activities physiologically trigger Mg^2+^ influx in neurons [182]. The action-potential-triggered Mg^2+^ signal apparently coordinates chemical and electrical synaptic activities and contributes to synaptic plasticity and formation of neural network.

### 4.2. Neural Cell Fate Determination 

Magnesium-L-threonate (MgT), which elevates the levels of Mg in the CSF of the brain, increases the numbers of neural stem cells (NSCs) in the hippocampus [29,183]. Mg^2+^ promotes the differentiation of NSCs to neurons instead of glial cells in vivo, while it promotes the differentiation to glial cells, not to neurons, in vitro [184,185]. In the rat brain, the Mg levels of all regions decline after postnatal day 5 [186]. Consistently, during the development of the mammalian nervous system, NSCs differentiate into neurons and glia in that sequence [187]. In addition, the activation of NMDA receptors increases the rate of oligodendrocyte differentiation via PKC activation [188]. In early development, changes in [Mg^2+^] around NSCs affect differentiation to oligodendrocytes via the Mg^2+^ modulation of NMDA receptor activities. Furthermore, TRPM7 channels may play a critical role in the proliferation and migration of astrocytes via the extracellular signal-regulated kinase (ERK) and c-Jun N-terminal kinase (JNK) signaling pathways [189]. Mg^2+^ influx is required for ERK activation in developing neuronal cells [38]. Therefore, TRPM7-mediated Mg^2+^ influx plays crucial roles in the proliferation and differentiation of neuronal cells.

## 5. Neuropathology of Mg^2+^ Homeostasis

### 5.1. Parkinson’s Disease 

PD is a neurodegenerative disease characterized by clinical symptoms, including tremors and rigidity. As almost 85 to 90% of the patients are sporadic, and 10 to 15% are familial, PD is believed to be caused by genetic and environmental factors [190]. PD pathologically shows the selective loss of dopaminergic neurons and the formation of Lewy bodies in the substantia nigra of the brain [190,191]. In cellular pathology, dopamine metabolism, mitochondrial oxidative stress, impaired protein degradation systems, and neuroinflammation are widely believed to be attributed to the selective death of dopaminergic neurons [191,192]. The brains of PD patients exhibit low concentrations of Mg in CSF [193]. Epidemiological studies revealed that the high incidence of PD is attributed to nutritional deficiencies of Mg^2+^ [194,195,196]. Continuous low Mg intake over generations damages mitochondria, ER, ribosomes, and nuclear DNA, as well as induces the loss of the dopaminergic neurons in the substantia nigra [8]. In some familial PD patients, a mutation in Mg^2+^-transporting proteins, e.g., TRPM7 [197,198] and SLC41A1 [199,200], has been reported. During the development of zebrafish, TRPM7 is essential for the production or release of dopamine in dopaminergic neurons [201]. Dietary Mg^2+^-deficit mice are susceptible to the toxicity of MPTP, which is a chemical inducer of PD [202]. The administration of Mg^2+^ inhibits the MPP^+^ neurotoxicity to dopaminergic neurons [203]. In the PD model of pheochromocytoma (PC12) cells, MPP^+^ induces the release of Mg^2+^ from mitochondria and the influx of Mg^2+^ across the cell membrane [39]. The suppression of Mg^2+^ influx decreases the viability of MPP^+^-exposed cells, and cell viability is highly correlated with [Mg^2+^]_cyto_ [39]. Moreover, the MPP^+^-induced inhibition of mitochondria itself altered the expression levels of cellular Mg^2+^-transporting proteins [168]. A 6-hydroxydopamine (6-OHDA)-induced PD animal model revealed lower levels of the SLC41A1 expression [204] and Mg^2+^ [205] compared with control rats.

α-Synuclein is a presynaptic neuronal protein that is pathologically linked to PD [191,192]. The aggregation of α-synuclein is considered to exert deleterious effects on the mitochondrial function [192,206]. Mg^2+^ at physiological levels directly inhibits the aggregation of α-synuclein, which is strongly promoted by other metal ions [207,208], suggesting that the interaction of Mg^2+^ and α-synuclein suppresses aggregation, and hence, neurotoxicity [209]. In addition, Mg^2+^ may inhibit the aggregation of α-synuclein by an indirect mechanism. Autophagy is a mechanism that transports misfolded protein aggregation and damaged organelles to the lysosome for degradation. The activation of autophagy promotes the clearance of cytoplasmic aggregated protein, including α-synuclein [210]. Thus, the impairment of basal autophagy causes abnormal accumulation and protein aggregation [211,212], and consequently, pathological features of PD in dopaminergic neurons [213]. mTOR signaling negatively modulates autophagy [214] and balances anabolism and catabolism in response to environmental conditions [215]. Thus, mTOR signaling affects the pathology of PD [216]. Although it is still controversial whether mTOR activity is neuroprotective or neurotoxic, the regulation of the mTOR signal is tightly connected to PD pathology via autophagy regulation [217]. Since intracellular Mg^2+^ is a regulator of mTOR signaling [38,116,128], the dependence of mTOR signaling on Mg^2+^ provides one explanation for the relationship between Mg^2+^ and the PD pathology. Such Mg^2+^ roles are expected to contribute to the protection of dopaminergic neurons in the substantia nigra from degeneration in concert with the other physiological roles of Mg^2+^, such as the suppression of ROS activities and the regulation of energy metabolism (described above).

### 5.2. Alzheimer’s Disease and Cognitive Functions

AD is the most common form of dementia in the population over 65 years old. AD is characterized by pathological features, such as hyperphosphorylated tau and extracellular senile plaques [218,219]. Senile plaques primarily comprise amyloid β (Aβ), and the accumulation of Aβ leads to the degeneration of neurons and resultant brain atrophy. Compared with healthy people, AD patients exhibit lower [Mg^2+^] in the CSF [198,220] and brain [220,221,222]. AD patients with lower [Mg^2+^] in the serum are likely to show more severe symptoms [223]. Mg^2+^ deficiency causes emotional memory dysfunction [224,225]. Mg^2+^ administration improves learning and memory in dementia patients [222] and in healthy animals [30,31,226] and promotes the recovery of cognitive function after brain injury [227,228]. In pathology, Aβ is sequentially cleaved from amyloid β precursor protein (APP) by β-secretase and γ-secretase. In contrast, α-secretase then cleaves APP into the C terminal fragment α (CTFα) and soluble APPα (sAPPα), which is neurotrophic. Since CTFα and sAPPα are elevated under high extracellular [Mg^2+^] conditions, the accumulation of the C terminal fragment β (CTFβ) and Aβ occur under low extracellular [Mg^2+^] conditions [229]. The elevation of extracellular [Mg^2+^] prevents the Aβ-induced reduction of the synaptic NMDA receptors via the suppression of the calcineurin overactivation in hippocampal slices [29]. MgT treatment reduces soluble APPβ and CTFβ, leading to Aβ aggregation and neuronal toxicity, which in turn prevent cognitive deficits and synaptic loss in the AD model of transgenic mice [29]. Extracellular Mg^2+^ in BBB reduces the influx of Aβ from blood to ECF and promote clearance of Aβ [19]. Furthermore, the treatment of MgSO_4_ attenuates impairments in long-term potentiation (LTP), dendritic abnormalities, and the impaired recruitment of synaptic proteins via the inhibition of glycogen synthase kinase-3β (GSK-3β) and activation of the PI3K/Akt signaling in sporadic AD model rats [230]. The inflammation is triggered by Aβ oligomers at the early stage. MgT decreases the TNF-α expression, which is a key mediator of inflammation, in glial cells and the expression of presenilin enhancer 2 and nicastrin, which are potential promoters of the Aβ synthesis, in neurons via a PI3K/Akt and nuclear factor-kappa B (NF-κB)-dependent mechanism [231]. Taken together, Mg^2+^ influx can suppress the proinflammatory mechanisms and protect neuronal functions in AD pathology.

### 5.3. Demyelination 

In demyelination mutant (*dmy*) rats, the loss-of-function mutation of mitochondrial Mg^2+^ uptake gene, *Mrs2*, is identified. In *dmy* rats, an increased number of mitochondria and abnormal content of metabolites are observed [232,233]. These observations revealed an association between mitochondrial Mg^2+^ homeostasis and demyelination. Myelin increases the conduction velocity of the action potential and energy efficiency. Although the mechanisms of axonal pathology and demyelination are not yet completely understood, the mitochondrial dysfunction is considered to play a central role [234,235]. The dysregulation of mitochondrial Mg^2+^ homeostasis disrupts the ATP production via the shift of mitochondrial energy metabolism and morphology. The *Mrs2* knockdown sensitizes cellular tolerance against cellular stress [48]. In addition, Mg^2+^-exposed oligodendrocytes exhibit more resistance to a hypoxic-ischemic injury [236]. In *dmy* rats, metabolic abnormality appears to lead to the downregulation of aspartoacylase, which cleaves the acetate moiety for use in the syntheses of fatty acids and steroids for myelination [237]. This report is consistent with the observation by metabolomics that decreased *Mrs2* expression leads to the abnormal metabolism of fatty acids [48]. In myelinating oligodendrocytes, double-strand breaks of mtDNA cause mitochondrial dysfunctions, consequently triggering demyelination and irreversible neurological deficit [238]. Mitochondrial Mg^2+^ affects mtDNA function and processes of mitochondrial central dogma independent of the exterior of the mitochondria. mtDNA lacks histones responsible for the formation of nucleosomes, and its absence causes the high rate of mtDNA mutagenesis (∼10-fold greater than in nuclear DNA) [239,240,241]. Thus, the roles of Mg^2+^ in DNA stabilization in mitochondria seem to be more dominant than that in the nucleus. Because the interaction of Mg^2+^ with DNA also contributes to the DNA/RNA stabilities [59], a low [Mg^2+^]_mito_ level should increase the risk of mtDNA damage. In addition, according to the endosymbiosis theory, mitochondria are endosymbiotic bacteria [242,243]. In bacteria, Mg^2+^ plays a key role in the regulation of protein synthesis [100]. Thus, mitochondrial Mg^2+^ may regulate protein synthesis in mitochondria. In fact, the separated regulation of mitochondrial and cytosolic protein synthesis in the process of the central dogma plays a central role in adaptation in the cellular nutrient environment [244].

## 6. Conclusions and Perspectives

Mg^2+^ is a versatile divalent cation because of its unique physicochemical properties. In the brain, the extracellular and intracellular Mg^2+^ levels dynamically change according to the biological context. Mg^2+^ plays crucial roles in cell proliferation, differentiation, survival, and neural network formation via the regulation of cellular metabolism, intracellular signaling, channel opening, protein synthesis, and ROS toxicity. Typically, the mobilization of intracellular Mg^2+^ stimulates catabolism and protein synthesis, consequently activating the cellular processes that determine the fate and phenotype. Although Mg^2+^ usually protects neuronal cells against cellular stress, excess levels of Mg^2+^ are sometimes deleterious to healthy neuronal functions. Therefore, the appropriate regulation of cellular Mg^2+^ homeostasis is essential for neuronal functions in the brain, and the dysregulation of Mg^2+^ homeostasis potentially causes and aggravates neurodegenerative diseases, such as Parkinson’s diseases, Alzheimer’s disease, and demyelination. The recovery of healthy Mg^2+^ homeostasis through chemotherapy targeting Mg^2+^-transporting system can improve cellular functions under pathological conditions. In summary, the regulation of Mg^2+^ homeostasis can be a candidate for a therapeutic target in neurodegenerative diseases. However, the roles of Mg^2+^ and its regulatory mechanisms have not been investigated much. Thus, further study is crucial for developing future therapies and deepening the understanding of its neuro(patho)physiology.

## Abbreviations

[Ca^2+^]	Ca^2+^ concentration
[H^+^]	H^+^ concentration
[Mg^2+^]	Mg^2+^ concentration
[Mg^2+^]_cyto_	cytosolic free Mg^2+^ concentration
[Mg^2+^]_ex_	extracellular free Mg^2+^ concentration
[Mg^2+^]_mito_	free [Mg^2+^] in the mitochondrial matrix
[Mg^2+^]_nuc_	nuclear free [Mg^2+^]
ACDP2	cyclin M2
AD	Alzheimer’s disease
APP	Aβ precursor protein
APPβ	β-amyloid precursor protein β
ATP	adenosine 5’-triphosphate
Aβ	amyloid β
BBB	blood–brain barrier
CNNM2	cyclin M2
CNS	central nervous system
CREB	cAMP response element binding
CSF	cerebrospinal fluid
CTFα	C terminal fragment α
CTFβ	C terminal fragment β
ECF	extracellular cellular fluid
ERK	extracellular signal-regulated kinase
GABA	gamma-aminobutyric acid
GSK-3β	glycogen synthase kinase-3β
IP3R	inositol-1,4,5-trisphosphate receptor
JNK	c-Jun N-terminal kinase
LTP	long-term potentiation
Mg	magnesium
Mg^2+^	magnesium ion
MgT	magnesium-L-threonate
mitoK_ATP_	mitochondrial ATP-sensitive potassium channel
MPP^+^	*N*-methyl-4-phenylpyridinium iodide
MPTP	1-methyl-4-phenyl-1,2,3,6-tetrahydropyridine
Mrs2	mitochondrial RNA splicing 2
mtDNA	mitochondrial DNA
mTOR	mammalian target of rapamycin
NF-κB	nuclear factor-kappa B
NMDA	*N*-methyl-D-aspartate
NSCs	neural stem cells
OGDH	2-oxoglutarate dehydrogenase
OXPHOS	oxidative phosphorylation
PC12 cell	pheochromocytoma cell
PD	Parkinson’s disease
PI3K	phosphatidylinositol-3 kinase
PKC	protein kinase C
PTP	permeability transition pore
ROS	reactive oxygen species
RyR	ryanodine receptor
sAPPα	soluble APPα
TCA	tricarboxylic acid
TRPM6	transient receptor potential melastatin 6
TRPM7	transient receptor potential melastatin 7
ΔG	Gibbs free energy change
ΔΨ_m_	mitochondrial membrane potential
